# Following Up on Clinical Recommendations in Transitions from Hospital to Nursing Home

**DOI:** 10.1155/2014/873043

**Published:** 2014-02-09

**Authors:** Lisa B. Caruso, Soe Soe Thwin, Gary H. Brandeis

**Affiliations:** ^1^Boston University Geriatric Services, Boston University School of Medicine, Boston Medical Center, 88 East Newton Street, Robinson 2, Boston, MA 02118, USA; ^2^Massachusetts Veterans Epidemiology Research and Information Center (MAVERIC), New England VA Healthcare System, 150 South Huntington Avenue, Boston, MA 02130, USA

## Abstract

Following up on recommendations made at the time of a hospital discharge is important to patient safety. While data is lacking, specifically around the transition of patient to nursing home, it has been postulated that missed items such as laboratory tests may result in adverse patient outcomes. To determine the extent of this problem, a retrospective cohort study of subjects discharged from an academic medical center and admitted to nursing homes (NH) was followed to determine the type of discharge recommendations and the rate of completion. In addition, for the purpose of generalizability, the 30-day hospital readmission rate was calculated. 152 recommendations were made on 51 subjects. Almost a quarter of the recommendations made by the hospital discharging team were not acted upon. Furthermore, for the majority of those recommendations that were not acted upon, a reason could not be determined. In concert with national data, 20% of the subjects returned to the hospital within 30 days. Further investigation is warranted to determine if an association exists between missed recommendations and hospital readmission from the nursing home setting.

## 1. Introduction

Transitional care is “a set of actions designed to ensure the coordination and continuity of healthcare as patients transfer between different locations or different levels of care in the same location [1].” Errors in transitional care can occur in several ways. Medication errors are made when a patient either receives the wrong medication at the destination site or an indicated medication is omitted from the discharge instructions. Another error that commonly occurs in transitional care is test follow-up error, that is, if a test is pending but not acknowledged in the discharge. In a study performed at a major teaching hospital in New York with a diverse patient population, 41% of the study participants with tests pending at the time of discharge had at least one test follow-up error [2]. A follow-up study found that, of recommended workups, 35.9% were not completed within six months of discharge [3]. Recently, Weiner and colleagues reported that half of the medical specialty referrals among an ambulatory population aged ≥65 were not completed by 180 days [4]. Another study found that physicians, including both inpatient and outpatient physicians, were unaware of 61% of abnormal test results that were considered “actionable.” Yet, they were unable to determine if these “missed results” were associated with adverse patient outcomes. These investigators noted that failure to follow-up test results may account for one-quarter of diagnosis-related malpractice cases [5]. These investigators and others recommend further work to design better follow-up systems [6]. The American Medical Directors Association (AMDA) has responded by formulating an 80-page online document entitled “Transitions of Care in the Long-Term Care Continuum” that includes a universal transfer form. This form includes a page for tests and appointments. All these initiatives are designed to improve patient care as the patient moves in and out of various healthcare settings [7].

As the patient moves from site to site, the opportunities for neglecting communication or to follow up on unresolved medical problems rise dramatically [8]. While the standard of care is for a discharge summary to accompany the patient when leaving the hospital and transitioning to NH, this document has been known to be missing or incomplete on arrival [9]. Were and colleagues reviewed hospital discharge summaries from two Midwestern hospitals with computerized electronic health records (EHR) and found that the discharge summaries were inadequate at documenting tests with pending results and the appropriate follow-up [10]. Several authors have suggested returning to handwritten forms or checklists to aid in communication when a patient is transferred from one site of care to another [1].

When a person is discharged from the hospital, a discharge summary is prepared. Today it is universally typed. It may be dictated or entered into the computer by the hospital discharging team. Generally, the discharging team is composed of a physician (attending or resident) and may also include a nonphysician practitioner (nurse practitioner or physician assistant) and/or nurse. It is their responsibility to list recommendations for future care of the patient to the provider in the next site of care.

For this study, the next site of care was the nursing home, where the physician and/or the nonphysician practitioner reviewed the recommendations to determine future actions. Studies examining specific items that are missed from transition from acute care hospital to nursing home are lacking. To our knowledge, the only research examining this transition was published by Gandara and colleagues who found that 47.2% of discharge packets from one healthcare system were missing the mention of pending test results. Gandara examined only the discharge documentation that was generated by the acute care hospitals [11]. To date, there are no studies which assess the completion of follow-up items through the entire episode of the transition of care in the NH.

The purpose of this study was to determine the number and types of follow-up recommendations generated during an acute care admission documented in the EHR that were completed within 180 days of hospital discharge to a NH and while the subjects were in the NH. Reasons for not completing the recommendations were also investigated. A secondary purpose was to determine the hospital readmission rate. Even though our study was too small to make any association between missed follow-up recommendations and hospital readmissions, reporting the 30-day hospital readmission rate adds to the generalizability of the study.

## 2. Design and Methods

### 2.1. Subjects

A medical record review was performed retrospectively on a cohort of patients discharged to nursing homes. Subjects were eligible if they were ≥65 years old and had been discharged from any medical or surgical inpatient service at Boston Medical Center, Boston, MA (BMC), between January 1, 2007, and June 30, 2007, and admitted to one of ten nursing homes (skilled nursing facilities/nursing facilities) in the greater Boston, MA area, serviced by Boston University Geriatric Services. Subjects were excluded if they were admitted to the nursing home for hospice care or if their records or charts were not available at the NH at the time of record review (i.e., charts had already been sent to off-site storage). All patients that met enrollment criteria were entered into the database. Using SAS 9.1, ten subjects from each month of the six-month study period were randomly selected to establish a convenience sample of sixty patients. Subjects were followed for 180 days after hospital discharge or until they were discharged from the NH, died, or returned to the acute hospital and did not return to the NH.

### 2.2. Data Collection

A data collection form was adapted from Moore et al. [3]. The original Moore form was tested for interrater reliability and found to have high reliability (*κ*, 0.89). The inpatient record, the discharge summary, and nursing home records were reviewed by study investigators (LBC, GHB) and four geriatric nurse practitioners. All clinicians extracting data were trained on completion of the data collection form. Inpatient medical records were either available electronically via Sunrise Clinical Manager system or available online as part of BMC's archival medical record system, GRID (Synergize-Streamlined Document Management). The medical provider notes from NH visits were hand-written in the nursing home record or available electronically as part of GE Centricity EHR. The data were collected electronically via a Microsoft 2003 ACCESS based software programmed with range and logic checks to ensure efficient and accurate electronic data entry. Once the data were entered into the database, all subjects were given a deidentifying number to ensure anonymity.

### 2.3. Analytic Variables

Data collection included demographic characteristics of the subject (age, race, ethnicity, and insurance), length of stay in the NH, and recommendations made during the inpatient stay including the category of recommendation (diagnostic procedures, subspecialty referrals, laboratory tests, physical exam reviews, and medication changes or monitoring), completion status of the recommendations, and reasons documented in the medical record describing why recommendations were not completed. Subspecialty referrals included appointments that were recommended to be made for neurology, endocrine, cardiology, psychiatry, gastroenterology, and general and surgical subspecialties. Laboratory tests included recommendations for basic metabolic panels, complete blood counts, PET scans, pending pathology reports, and urine culture results. Medication changes or monitoring included duration of antibiotic treatment, monitoring levels of medications, deep vein thrombosis prophylaxis, and titration of medications.

### 2.4. Data Analysis

The data were analyzed using SAS 9.1. Summary statistics describing the pattern of recommendation completeness were generated. The protocol was approved by the Boston University Medical Campus Institutional Review Board.

## 3. Results

There were 222 subjects ≥65 years old at the time of discharge from Boston Medical Center, Boston, MA, between January 1, 2007, and June 30, 2007, who were admitted to one of ten nursing homes serviced by Boston University Geriatric Services. Sixty records were consecutively reviewed to obtain the analytic sample of 51 subjects. Four subjects were not admitted to the NH from BMC, four subjects had incomplete records, and one subject had a duplicate medical record number so could only be used once. No subjects were on hospice at the time of NH admission.  Table 1 shows the baseline characteristics of the study sample. The average age was 80.6 ± 9.3 (range 65–106). The sample was mostly white women with Medicare as the primary payer source. The length of stay at the hospital was 6.1 ± 3.4 days (range 1 to 16 days) prior to NH admission. The majority of the subjects were discharged from the geriatrics (36%), general internal medicine (32%), and family medicine (4%) services. 16% were discharged from a surgical service. The remainder were discharged from other specialty services such as neurology or oncology.

Table 2 lists the categories of recommendations made for the 51 subjects. A total of 152 recommendations were made by inpatient providers. The most common type of recommendation was a subspecialty referral followed by laboratory tests and medication changes or monitoring. Of the 152 recommendations made, 37 (24%) had no documentation of follow-up within six months of the hospital discharge. 32 of these recommendations were identified from the discharge summary, and 5 were found on review of the inpatient hospital record (GRID and Centricity).

Table 3 lists the categories of recommendations that were not completed within 180 days of hospital discharge. Of those 37, the receiving NH provider team documented reasons for declining 13 (35%); no reason was documented for the remaining 24 (65%).

The number of follow-up recommendations for each subject varied (range 0–8) with the mode being 2. As the number of recommendations made increased, the number followed decreased. For those subjects with one, two, and three to eight follow-up recommendations, 88%, 53%, and 23% were completed, respectively. During the study period, 23 of 51 subjects (45%) had hospital readmissions during the study period. 10 of these 51 returned within 30 days (20%).

## 4. Discussion

We tracked recommendations made during acute care hospital inpatient stays for subjects discharged to a NH. Almost a quarter of recommendations made during the inpatient stay were not followed after the transition of care. The three most common recommendations not followed in our study were (1) subspecialty referrals, (2) laboratory tests, and (3) medication changes/monitoring.

While, to our knowledge, there are no studies similar to ours for comparison, it may be instructive to look at the findings of Moore and colleagues who examined discharges from hospital to community [3]. As expected, Moore's sample was younger (average age of 58) [3]. Our finding of 24% of recommendations not completed compares to Moore's finding of 35.9% of recommended workups not completed after hospital discharge. Our rate was likely lower because there was a dedicated team that saw the patient within a week of discharge and who were more attuned to following up outstanding items due to the higher vulnerability of patients being admitted to the nursing home.

Moore found that the three most common categories of recommendations not followed were (1) diagnostic procedures, (2) subspecialty referrals, and (3) laboratory tests. Diagnostic procedures were the least common type of recommendation needing follow-up in our study (4.6% of all recommendations). This difference may be due to the fact that our sample of older, more frail NH subjects may have been thought to need fewer diagnostic procedures as opposed to outpatients in the Moore study [3]. It is also possible that diagnostic procedures were easier to obtain at BMC, and so they were able to occur during the acute hospitalization.

It has been estimated that 20%–33% of older patients are rehospitalized within 30 days [12]. Our results are consistent with these figures. However, our sample represents a select, frail population. One may posit that the rate at which subjects were readmitted may have been higher if not for the EHR and the single medical practice within one medical center that cared for these NH residents. Strengths of the study include the use of an integrated EHR. Accepting providers at the NH had access to all documentation from the acute care hospitalization.

The current study has several limitations. First, the sample size was small. It is a single site study from a safety net hospital with an integrated geriatrics practice. Due to time and resource constraints, only 60 discharges were selected and 51 were reviewed. In addition, we were unable to compare the qualities of our small sample to the unselected population. However, our randomization method should mitigate any differences between the groups. Second, while the acute care portion of the record was electronic, all NHs used paper records. Third, while we were able to determine the readmission rate of the study population, because of the cross-sectional design of the study, we were limited in our ability to relate readmissions to noncompletion of recommendations. Lastly, tracking of pending lab tests for most subjects was dependent on what was documented in the discharge summary and the inpatient progress notes. From the recent study published by Walz and colleagues, 32% of the patients discharged from acute care to subacute care were discharged with pending lab tests alone, not including any other type of recommendation [13].

A question remains for future investigation: does a relationship exist between noncompletion of inpatient recommendations and hospital readmissions? While the present study was not able to determine an association, there does seem to be face validity to the premise that missed recommendations would lead to hospital readmissions especially in patients with multiple comorbidities and functional impairments.

As has been previously observed, many inpatient recommendations are not followed. Specifically, in our study, the rate of nonfollow-up is less than the others have found. We believe that the use of an EHR is a major reason. The receiving team at the NH had access to the inpatient record. In most cases, the receiving provider also had access to the entire outpatient record prior to the acute hospitalization. We would not expect that all recommendations by the inpatient care team would be followed by the NH admitting team. The NH team providers have expertise in NH care and likely determine that some acute care recommendations are not justified. Other potential reasons why some acute care recommendations were not followed could have been (1) the NH providers had a reason for not following the recommendation, such as a change in the goals of care, but did not document it, (2) the NH providers missed the recommendation, or (3) the patient was discharged from the NH before the recommendation could be acted upon.

Since this study was completed, several hospital wide initiatives have been instituted at BMC to decrease any missed recommendations. First, all future primary care and specialty care appointments are automatically entered into the discharge summary. Second, the names of the members of the discharging team, including the attending and house staff, are listed along with the outpatient primary care provider. Third, any medication that was added or changed during the hospitalization is documented on the discharge summary, thus reconciling the prehospital medication list with the discharge medication list. Fourth, a separate section titled “outstanding issues” has been added to prompt the house staff to enter items for follow-up. However, the electronic discharge summary does not automatically download any outstanding laboratory values at the time of discharge.

Nationally, other efforts are being made to improve transitional care, given the currently limited interoperability between EHRs in various sites of care. Interact II is an initiative to “improve care and reduce the frequency of potentially avoidable transfers to the acute hospital [14].” The Resident Transfer Form, found in the Interact II Tool Kit, provides a medical summary, a reason for transfer as well as cognitive and functional baselines, active behavioral and social issues, and important contact numbers. Adopting standardized approaches to transfers helps ensure that important issues needing follow up are communicated to receiving providers. There is early evidence that standardizing the transitional care process using Interact II leads to a decrease in avoidable hospitalizations from NH [15]. Perhaps the rate of unresolved medical recommendations would decrease as well.

Boockvar and Burack have found that management-level organizational relationships between nursing homes and hospitals are associated with more optimal transfers [16]. While the NHs in our study were not affiliated with BMC, they did provide Internet connections so that BMC providers could access BMC's EHR. Improved organizational relationships between NHs and hospitals will help to efficiently and cost-effectively provide transitional care. As NHs purchase EHRs, it will be important that they are compatible with those of the hospitals. As we leave the era of not having information because it was not written down or communicated, we risk entering the era of having the data but being unable to access it.

The majority of hospital discharge recommendations were followed in the NH, yet a quarter were not. The reasons why are only partially understood. While organizations such as AMDA have written guidelines to help with transitions, much work needs to be done to operationalize the discharge process [7]. An EHR that is accessible across key sites of care will be an important component.

## 5. Conclusions

Almost a quarter of the recommendations for subjects discharged from an acute care hospital to a nursing home are not followed after transitions of care. Noncompletion was associated with an increasing number of recommendations, lack of documentation in the discharge summary, and the type of recommendation. Establishing better processes of communication during transitions of care may help to reduce noncompletion of recommendations and enhance their appropriateness. One process that was instituted at BMC added a formal section “outstanding issues” to the discharge summary. Further work needs to be done to allow EHR to automatically add “outstanding issues” to the discharge transfer form.

## Figures and Tables

**Table 1:** Baseline characteristics of the study sample (*N* = 51).

Characteristic	Frequency (%)
Age in yrs (mean ± SD)	80.6 ± 9.3
Race	
White	25 (49)
Black	23 (45)
Hispanic	3 (6)
Gender	
Female	42 (82)
Male	9 (18)
Language	
English	34 (67)
Non-English	17 (33)
Insurance	
Medicare	42 (82)
Medicaid	2 (4)
Medicare managed care	3 (6)
Medicare advantage (i.e., senior care options)	3 (6)
Other	1 (2)
Hospital length of stay in days (mean ± SD)	6.1 ± 3.4

**Table 2:** Categories of recommendations made by the hospital discharging team.

Type of procedure	Frequency (%)
Diagnostic procedures	7 (4.6)
Subspecialty referrals	46 (30.3)
Laboratory tests	36 (23.7)
Physical exam reviews	15 (9.9)
Medication changes/monitor	27 (17.8)
Other	21 (13.9)

Total number of recommendations	152

**Table 3:** Types of recommendations not pursued by 6 months following hospital discharge.

Recommendations	Total number of recommendations not completed (%)
Diagnostic procedures (%)	3 (8)
Subspecialty referrals (%)	13 (35)
Laboratory tests (%)	9 (24)
Physical exam (%)	2 (5)
Med changes (%)	6 (16)
Other (%)	4 (11)

Total	37
